# Exploring self-reliance in primary healthcare from peoples’ perspectives among families using traditional medicine: A case study from southern India

**DOI:** 10.36368/jcsh.v2i1.1207

**Published:** 2025-10-26

**Authors:** Shivanand Savatagi, NS Prashanth, Unnikrishnan Payyappallimana, John DH Porter, Upendra Bhojani, Harini Nagendra

**Affiliations:** 1 The University of Transdisciplinary Health Sciences and Technology, Bengaluru, India; 2 Center for Health Systems, Institute of Public Health, Bengaluru, India; 3 London School of Hygiene and Tropical Medicine, London, England; 4 Azim Premji University, Bengaluru, India

**Keywords:** Self-reliance, primary Healthcare, traditional medicine, people-centered health system, self-care, Autosuficiencia, atención primaria de salud, medicina tradicional, sistema de salud centrado en las personas, autocuidado

## Abstract

**Introduction:**

Self-reliance consists of using available resources, making informed decisions, and responsible health and lifestyle practices. The complex nature of healthcare practices by the people necessitates the quest for understanding self-reliance in primary healthcare. In LMICs, around 70% in India and approximately 80% in sub-Saharan African countries use traditional medicine for primary healthcare. Because of its emphasis on local resources, context, culture, and other determinants, traditional medicine potentially enables self-reliance in primary healthcare. The study explored the meaning of self-reliance in primary healthcare from peoples’ perspective among families using traditional medicine in a Southern Indian site.

**Methodology:**

We conducted case study research in HD Kote, a taluka in Mysore district of Karnataka in Southern India, from September 2021 to April 2022. We selected 30 study participants using purposive sampling and the data was collected through in-depth interviews. Data was subjected to thematic analysis using QDA Miner lite 2.01 version software.

**Results:**

The study identified following themes: 1) perception of health and illness, 2) healthcare pathways, 3) negotiation between the environment and self in the emergence of self-reliance, and 4) efficiency and authenticity as a means to choose healthcare approach. Pathways to healthcare decisions were influenced by various cultural, social, religious, health system, and individual level factors. Self-reliance emerges from an individual through negotiation between the self, the environment, and the social context in which they live.

**Conclusion:**

The meaning of self-reliance was navigated within healthcare practices, choices, and the context in which an individual lived. Capturing people’s perspectives and local views addresses the gap in scientific understanding by situating these perspectives within the broader context of the health system, particularly in relation to self-reliance and the role of individuals. Understanding self-reliance through studies of this kind is critical to contributing to a people-centered health system for India to achieve the goal of universal health coverage.

## Abstract (es)

**Introducción:**

La autosuficiencia consiste en utilizar los recursos disponibles, tomar decisiones informadas y mantener prácticas responsables de salud y estilo de vida. La naturaleza compleja de las prácticas de atención sanitaria de las personas exige profundizar en la comprensión de la autosuficiencia en la atención primaria de salud. En los países de ingresos bajos y medianos (LMIC, por sus siglas en inglés), alrededor del 70% de la población en India y aproximadamente el 80% en los países del África subsahariana utilizan la medicina tradicional como parte de la atención primaria de salud. Debido a su énfasis en los recursos locales, el contexto, la cultura y otros determinantes, la medicina tradicional tiene el potencial de fomentar la autosuficiencia en la atención primaria. Este estudio exploró el significado de la autosuficiencia en la atención primaria desde la perspectiva de las personas, entre familias que utilizan la medicina tradicional en una región del sur de la India.

**Metodología:**

Se realizó una investigación de estudio de casos en HD Kote, un taluka (distrito) de Mysore, en Karnataka, al sur de la India, entre septiembre de 2021 y abril de 2022. Se seleccionaron 30 participantes mediante muestreo intencional, y los datos se recolectaron a través de entrevistas en profundidad. La información se analizó temáticamente utilizando el software QDA Miner Lite versión 2.01.

**Resultados:**

El estudio identificó los siguientes temas: 1) percepción de la salud y la enfermedad, 2) trayectorias de atención sanitaria, 3) negociación entre el entorno y el yo en la aparición de la autosuficiencia, y 4) eficiencia y autenticidad como criterios para elegir el enfoque de atención sanitaria. Las trayectorias hacia las decisiones de atención en salud estuvieron influenciadas por diversos factores culturales, sociales, religiosos, del sistema de salud e individuales. La autosuficiencia surge de la persona a través de la negociación entre sí misma, su entorno y el contexto social en el que vive.

**Conclusión:**

El significado de la autosuficiencia se manifiesta dentro de las prácticas de atención en salud, las elecciones individuales y el contexto en que la persona habita. Captar las perspectivas y visiones locales de las personas permite abordar las brechas en la comprensión científica al situarlas dentro del contexto más amplio del sistema de salud, especialmente en relación con la autosuficiencia y el papel del individuo. Comprender la autosuficiencia a través de estudios de este tipo es fundamental para contribuir a un sistema de salud centrado en las personas y avanzar hacia el logro de la cobertura universal de salud en la India.

## Introduction

The capacity and ability of an individual to make informed health decisions and to use available health resources are crucial components for the effective prevention and management of health conditions. Autonomy and personal agency are important factors determining health outcomes [1]. Individual capacities and abilities notwithstanding, a substantial proportion of mortality from the leading causes of death (e.g., cardiovascular diseases and cancer) is due to influence of social determinants on health. The morbidity and mortality pattern from such diseases (cardiovascular disease, hypertension, diabetes, and cancer) is preventable and modifiable through changing lifestyles, making right healthcare choice, healthy dietary habits, and increasing the health literacy of individuals [2]. Practice of healthy lifestyle, healthy dietary habits, and informed healthcare choice are called by different terminologies in health literature that include self-care, self-reliance, self-efficacy, and self-management. Although these terms have slight differences, they are interconnected. Self-care, self-efficacy, and self-management are all related to the process of achieving self-reliance. Savatagi et al differentiate these terms; self-care is an attitudinal component where an individual trusts his/her ability towards action, self-efficacy is the confidence through which an action is carried out to manage a health condition and self-management is the ability to manage unanticipated consequences in the process of an action [3]. Self-reliance is a state of mind an individual reaches after acquiring self-management skills where one trusts and believes in their skills. Thus, self-reliance encompasses all other terms in it and their common focus remains on enhancing the use of local resources and the individual’s responsibility towards one’s health [3,4]. The interchangeable use of different terms (self-care, self-efficacy, self-management, and self-reliance) in the health literature reflects a lack of clarity in their conceptualization in relation to peoples’ understanding that has led to confusion around integrating self-care, self-reliance, self-efficacy, and self-management as a strategy for health systems strengthening [3]. We used the term ‘self-reliance’ in this study since it encompasses all the other terms as discussed by Savatagi et al. [3].

Since the 1970s and 80s, self-reliance has been a significant theme of discussions and debates in primary healthcare and it is the most commonly used term in the global policy documents in the context of primary healthcare [5]. However, to implement health interventions that aim to enable self-reliance of people in primary healthcare, there is a need to understand how self-reliance emerges within complex healthcare practices, choices, and healthcare decisions that shape health behavior in household settings. Primary Health Care (PHC), as articulated in the Alma-Ata (1978) and Astana (2018) Declarations, represents a broader systems-level philosophy that emphasizes equity, participation, and intersectoral collaboration across sectors [6,7]. Because self-reliance is evident operationally at the family, and community level by the people, this paper begins its inquiry from that sphere to explore how everyday practices, choices, and community actions collectively shape the realization of self-reliance within the broader vision of PHC [8].

India’s healthcare system is a pluralistic mix of public, private, traditional medicine, and community-based providers. The public sector follows a three-tier structure, from primary health care (sub-centres and primary health centres) to secondary (district hospitals) and tertiary (specialty hospitals), with state governments leading service delivery [9]. Alongside, a large and diverse private sector, from hospitals to informal providers, plays a dominant role, while community health workers such as the Accredited Social Health Activists (ASHAs) link households to essential services [10,11]. There are traditional medicine practitioners who deliver services at the community level. This complex mix creates multiple points of access but also challenges of fragmentation, variable quality, and inequities [12]. Traditional medicine (TM) faces challenges related to quality assurance, standardization, and evidence-based validation, with concerns about safety and efficacy of certain practices [13–16]. Nonetheless, TM remains central to household healthcare and primary care in India, offering affordable, accessible, and culturally acceptable care, and continues to be recognized by the World Health Organization (WHO) as a vital component of primary health care [17–19].

Primary health care is an important organizing principle for health systems with the potential to strengthen the health system through a focus on promotion, prevention, and a people-centered approach. Despite a significant investment in primary health care, the challenges of availability, accessibility, affordability, and a lack of responsiveness of the health system [20–22] persist. Hence, integrative approaches, i.e. healthcare approaches that aim for coordinated care among different healthcare providers and institutions by bringing modern medicine and TM together to care for the whole person [23] while ensuring safety and efficacy, have been advocated [24]. Indeed, a primary health care system where people have the option to choose different healthcare approaches which are known to be safe and effective, with or without becoming dependent on institutionalized medical approaches could improve people’s health.

In low- and middle-income countries (LMICs), a significant proportion of common ailments are treated by the individual or the family without professional intervention using traditional or modern medicine interventions [25,26]. This contributes to a substantial proportion of primary healthcare interventions [27]. For instance, in India, around 70% and approximately 80% of sub-Saharan African countries use TM for primary health care [28,29]. Because of its emphasis on local resources, context, culture, and other determinants, TM is likely to contribute to self-reliance for primary health care [28,30]. The attributes of self-reliance are using locally available resources, making informed decisions, and responsible health and lifestyle practices [31].

TM encompasses codified and non-codified systems [32]. The codified systems have acquired legitimacy and formal recognition. Examples include Ayurveda, Yoga, Unani, Siddha and Homeopathy in India. Non-codified or folk medicine is an inter-generational oral knowledge system existing in several communities. In the Indian setting, this includes bone setters, birth attendants, local healers, and the use of home remedies by mothers or elders in the family for addressing common illnesses [33,34]. These healthcare practices are adopted at the individual level, within household and neighborhoods, and across entire communities with variations from region to region. Eventually, a people-oriented public health practice will need to understand how people choose, practice, and perceive different healthcare approaches to address their health problems [27]. This could nurture a more meaningful integration of TM with safety and quality guidelines that strengthen self-reliance in primary healthcare [5,33]. Self-reliance could be a strategy that contributes to a people-centered approach since it endorses building peoples’ capacities to address their health problems [35].

In India, the *Ayushman Bharat* health and wellness program aims to transform existing primary healthcare centers and sub-centers into health and wellness centers. Health and wellness centers address health issues at the community level to reduce the burden on higher level health facilities [36]. Going further, the AYUSH ministry proposed to upgrade 12,500 health and wellness centers to AYUSH health and wellness centers to establish a holistic wellness model based on AYUSH principles and practices and to empower people on self-care to reduce disease burden, out-of-pocket expenditure, enable informed choice, and use medicinal plants for treating common ailments [37]. The broad aim of these initiatives is to enable the self-reliance of families and communities in primary healthcare. However, the limitation of the *Ayushman Bharat* program is that the program does not mention people as part of the healthcare delivery network but rather emphasizes channelizing delivery of services through community health workers (ASHA, community health officer, and Anganwadi workers) which makes people again passive receivers of healthcare [38] resulting in less engagement as active partners of their health. AYUSH health and wellness centres also continue to adopt the institutional-based approach resulting in the challenges of monitoring and conducting community-level activities due to manpower shortage [39]. These challenges reiterate the need for empowering people to self-care that enhances self-reliance in primary healthcare among families and communities.

This study draws upon fieldwork conducted in H D Kote *taluka* (an administrative sub-division of a district) of Mysore district in a state of southern India. It has a mix of rural, urban, and tribal populations and health J Community Systems for Health problems are diverse [40,41]. In addition to government efforts, several non-governmental organizations (NGOs) in the region have historically worked towards improving healthcare services. For instance, the mobile health unit of one of the NGOs, the *Swami Vivekananda* Youth Movement (SVYM) in Saraguru *taluka* [42], the promotion of TM by the Foundation for Revitalization of Local Health Traditions (FRLHT) – Bengaluru (now known as The University of Transdisciplinary Health Sciences and Technology (TDU), Bengaluru [43], and a community health worker approach by the Karuna trust, Bengaluru [44] are a few initiatives by NGOs often in partnership with the government. These initiatives started with the intention of enabling the self-reliance of families in primary health care. Hence, these initiatives provide an opportunity to conceptualize self-reliance from people’s perspective.

Various factors influence the manifestation of self-reliance at an individual level. For instance, experiential knowledge, embodied knowledge, and cognitive construction of disease significantly impact one’s healthcare decision [45]. These different forms of iterative knowledge are expressed within a specific context exhibiting an adaptive ability to manage a disease condition [45]. This ability contributes to an individual’s self-understanding of illness, self-care, self-control, and eventually how to self-manage the disease [3,46]. Given the multilevel manifestation of self-reliance, a research approach that understands the contextual factors through people’s experiences will be critical in conceptualizing and practicing self-reliance. Analyzing people’s perceptions and practices of healthcare within the local health systems context and navigating meaning of self-reliance in relation to primary health care and TM is a unique feature of this study. Hence, the aim of this case study was to explore the meaning of self-reliance in primary healthcare from peoples’ perspective among families using traditional medicine. This study is part of an effort to develop a self-reliance framework for primary health care using TM. The findings of this study were used to refine a self-reliance framework that evolved from a scoping review [3].

## Methods

### Study setting

H D Kote, a *taluka* in the Mysore district in Karnataka in Southern India, is surrounded by western ghats (a biodiversity hotspot in India) in the West and South [47]. The southern part of the *taluka* is also a part of the Nilgiri biosphere reserve [48]. The taluka is a rich source of medicinal plants due to its proximity to Nagarhole and Bandipur forests [49]. Many families and traditional healers use medicinal plants (*mane maddu* - home remedies in Kannada, the local language) for common health problems such as fever, cold, cough, body ache, vomiting, stomachache, dysentery, etc. [49]. Families reported several traditional health practices either through self-treatment, using medicinal plants (sourced Savatagi et al. from the wild or from forest neighborhoods), support from family members, and social groups [50]. In addition to the idea of classical medicines taken orally, rituals performed by the people or on their behalf, or consumption of food in specified ways, for instance to prevent diseases, or promote health are also widely reported self-care practices adopted at the household level in H D Kote [33,51]. The region reports a heterogeneous caste and tribal composition, including Scheduled Tribes (notably *Jenu Kuruba, Betta Kuruba*, and *Soliga*), Scheduled Castes, Other Backward Classes, and dominant caste groups such as *Vokkaligas* and *Lingayats*. These hierarchies influence access to land, education, and healthcare, with Scheduled Tribes and Castes often experiencing social and economic marginalization. In addition to this, culture, social norms, spiritual practices, moral behavior, and values remain integral parts of people’s health. Although communities in this region have transitioned towards modern healthcare practices like many other communities in Southern India, many families continue to rely on self-care practices rooted in their traditional medicine, inter-generational beliefs, traditions, and culture as well as in their newly acquired modern healthcare knowledge [52].

We conducted this study in H D Kote from September 2021 to April 2022. As per the census 2011 [53], H D Kote has a population of approximately 263,000. The majority of the population (95.29%) self-identified their religion as Hindu, followed by Islam (∼3%) and Christianity (1.05%). The average sex ratio of the taluka is 987 females per 1000 males. Most of the population (90.02%) live in rural areas. The Indian Constitution includes provisions to safeguard rights of communities that have faced socio-economic disadvantage and exclusion, the Scheduled Caste (SC) and Scheduled Tribes (ST). About 23% of the population belong to ST and 27.8% belong to SC. The total literacy rate is 64.11%. Kannada is a native language in villages and in a few villages that share borders with Kerala, people speak both Malayalam and Kannada. H D Kote has a mix of tribal, rural, and peri-urban contexts. The predominance of low-income occupations like farming, daily wage labor, and forest-based livelihoods contributes to fragile socioeconomic status, which limits households’ financial security. This directly affects healthcare accessibility and affordability, as irregular incomes often constrain choices, delay care-seeking, and increase dependence on low-cost or informal providers. Government and private health facilities deliver health services in the region.

### Study design

We conducted this study using a case study design. A case study method is used to generate an in-depth and multifaceted understanding of a complex issue in a real-life context. The case study method also offers an opportunity to learn from experiences and influence the practice of theories [54]. Since self-reliance is embedded in the experiences of individuals, we adopted the case study method to capture the experiences of people using TM to investigate the phenomenon of self-reliance.

### Sampling

A case was defined as a household practicing TM and residing in HD Kote. Each household possessing such characteristics is identified as a case. The selected households are referred to as cases (study participants). The cases were selected based on the criteria; 1) should be practicing TM in their household, 2) should be residing in HD Kote, and 3) should be willing to share experiences and knowledge on TM.

We purposefully selected a project that documented household knowledge of TM for minor health problems in H D Kote. The project was implemented by TDU in partnership with SVYM. Cases were selected using the purposive sampling. The TDU provided a list of households practicing TM in HD Kote. With this list, we approached a field coordinator of SVYM to seek help in identifying information-rich cases (those participants who were interested in sharing their experiences of using TM and other healthcare approaches). The field coordinator further sought the help of a health facilitator (HF) of respective villages to recruit participants. A health facilitator was responsible for a specific village covered by SVYM in their health intervention projects and stayed in or near the village. If HF was familiar with the village, she/he could trace the study participants. In situations where HF was new and was unable to trace, we sought the help of ASHA or Anganwadi workers (government community health workers and nutritional health service providers respectively) to identify a potential participant. We explained the study to the HF, ASHAs, and AWWs before seeking their help. Cases were selected in such a way that a mix of rural, tribal, and peri-urban participants were included.

**Figure 1:**
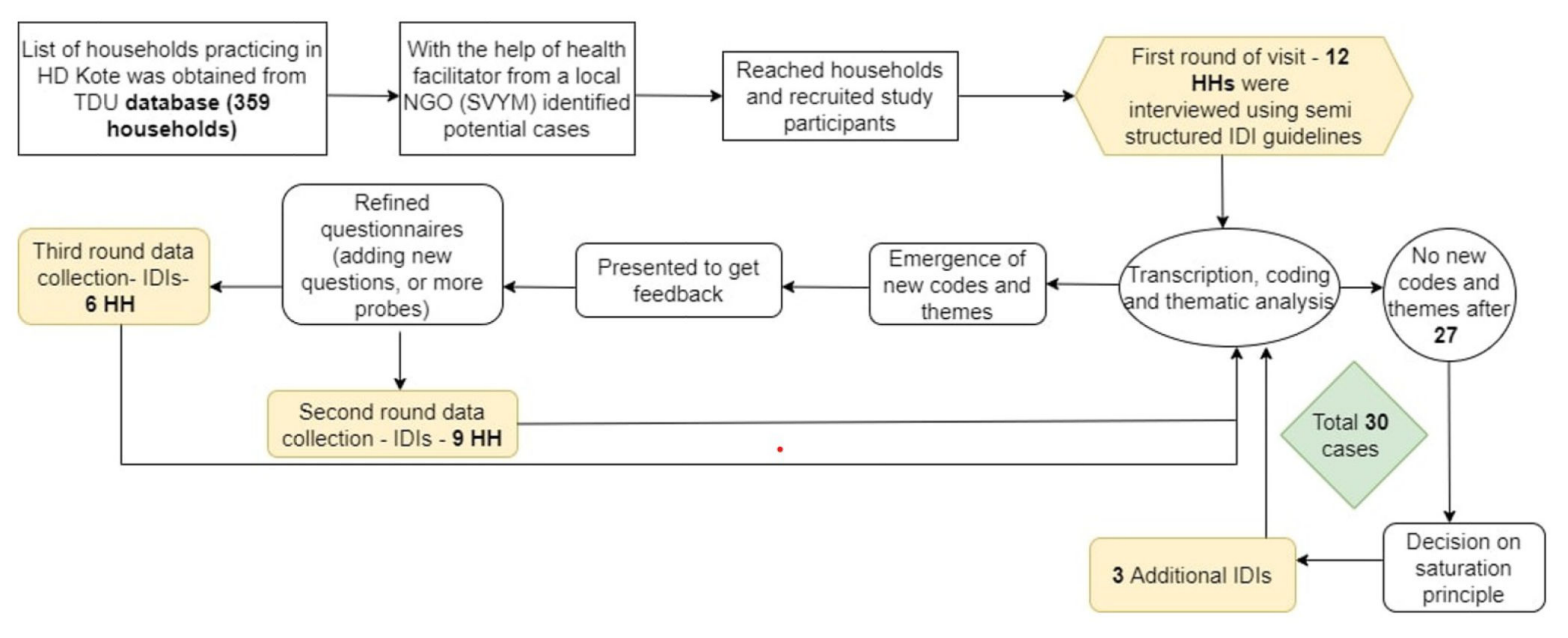
Replication logic used for determining the sample size.

In case-study research, replication logic is used as a sampling technique to obtain an adequate number of study participants [55]. Replication logic involves selecting a few cases initially with pre-defined criteria and collecting and analyzing data. Further cases were included until no new information emerged from the data. This logic also involves selecting contrasting cases to support or reject the initial theory [55]. From the secondary data of TDU, we observed that, out of 650 households surveyed in HD Kote, 359 households were using TM for minor health conditions. From this database, 30 cases were included purposively using replication logic (Figure 1).

### Data collection

We collected data using in-depth interviews (IDI) and direct observation. Two data collectors, an interviewer (first author), and a note-taker collected the data. Both data collectors were native speakers of Kannada and were not from H D Kote. Through purposive sampling, we collected data from 30 cases at the participant’s house or a place where the participant felt comfortable. After 27 cases, data was deemed repetitive, and we conducted an additional three interviews to confirm data saturation. We also encountered rejection from three participants as they did not wish to share TM knowledge. Of these three participants, two were younger participants: one male with graduate level education and one woman with high school level education. The third was an elderly tribal male with no schooling, who expressed that sharing knowledge outside the community was considered inappropriate, was against their cultural practice and could render it ineffective if shared with outsiders. We used direct observation technique as supportive documentation to enrich contextual understanding for the data collected through interviews. We observed the presence of medicinal plants surrounding participants’ houses, instances where patients sought care from traditional medicine, and the methods used in preparing traditional medicine. These observations were not analyzed as separate data but were used to triangulate and complement the interview findings, providing a deeper contextual background to the study. All interviews were saved on a recorder, and observations were noted on paper. We expanded the notes on the day of IDI to incorporate them during the analysis.

The first author developed the topic guides for collecting the data, and the second and third authors reviewed and suggested refinement in terms of adding more probes before finalizing the guides. The topic guides were pilot-tested before starting the data collection. The first author conducted interviews in Kannada and transcribed them. The second and third authors joined in cross-validation. Topic guides were refined as and when required during the data collection process, mainly in terms of adding more probes. Each interview or focus group discussion lasted for 30 min to one hour. The data captured was primarily about understanding health and illness, the healthcare approaches adopted by study participants, their inter-generational knowledge of TM and how they used it, practice of different healthcare approaches, and their reasons. The meaning of self-reliance was derived from their understanding of health, practices relating to health, decision process followed for the choice of healthcare approach, and their beliefs about their healthcare practices.

### Data management and analysis

All interviews were translated into English before exporting to the software for analysis. All the transcripts were read in front of the study participants for reconfirmation of the information. We used an inductive thematic analysis approach (56). The first author started analyzing the data simultaneously along with data collection and the second and third authors cross-checked the data validity. We used QDA Miner lite 2.01 version software for creating codes and themes [57]. The codes and themes were discussed periodically with the other authors and refined. The data analysis included capturing the experiences of study participants about the meaning of health, healthcare practice, systemic challenges of accessing healthcare, and cultural, religious, and other factors influencing their abilities to taking healthcare decisions. The analysis also focused on comprehending the meaning of local dialects to synthesize the meaning of self-reliance. We employed two key approaches to enhance the rigor and trustworthiness of our study findings. Member checking was carried out with the selected participants to confirm the findings are a true reflection of the discussions. We also carried out de-briefing with the research team and also with health systems research experts to receive their feedback [58].

### Positionality of the researchers

The first author, as a doctoral researcher, led the study. He identifies as a Hindu (religion) and grew up in a rural setting with Kannada as his first language. He gained knowledge about local health traditions as part of his earlier work at TDU, so he could relate and engage with the study respondents well. He acquired degrees in nursing and public health with formal training in qualitative and health systems research. While investigating the self-reliance concept he positioned himself as both an insider and an outsider, combining contextual familiarity with critical distance to offer a more nuanced perspective. As a Hindu, his worldview might have shaped certain community understandings of health and care-seeking, but analytical framing was guided by public health and health systems perspectives rather than religious, and cultural interpretations. Other authors are either Ph.D. supervisors or part of the Doctoral Advisory Committee of the first author. They all are public health professionals with expertise in health policy and systems research as well as researching local health traditions and contributed intellectually through critical inputs to the manuscript development.

### Ethical considerations

The study obtained ethical review clearance from the ethics committee for human research of TDU, Bengaluru (study protocol number: TDU/IEC/11/2020/PR38). We adhered to all the ethical guidelines during the study. Potential risks and benefits were explained to the participants before taking the consent. We obtained both oral and written consent from the study participants. For participants who were unable to read the consent form, a health facilitator from *Swami Vivekananda* Youth Movement (SVYM), who was fluent in local dialects and familiar with the community, read the consent form aloud and explained in a simple and culturally appropriate terms about the purpose of the study, procedures, potential risks and benefits, voluntary nature of participation, use of data, and the right to withdraw at any time without consequences. For participants unable to sign, thumb impressions were obtained in the presence of a witness, along with oral consent. The household list of TM practitioners used in this study was originally compiled by the TDU with institutional permission to document and develop databases of TM users in HD Kote, with the explicit understanding that such information could be used for research purposes related to TM practices. For this study also, we sought permission from study participants to record interviews, discussions, take pictures, document their knowledge of TM, and publish the study’s findings. Participants had an opportunity to continue or withdraw from participating in the study at any time in course of our visits to the site. The collected data was coded, anonymized, and confidentiality was maintained throughout the study. We labelled data files and names with the codes and nowhere was the identity of the respondent revealed. We restricted data access to research team only.

### Patient and public involvement

SVYM being a local NGO with a long track-record of community engagement, was involved in the selection of information rich cases during the process of data collection. Meetings and interactions with SVYM were held from the initial phases while the study was being conceptualized.

## Results

The meaning of self-reliance was interpreted through peoples’ health experiences, healthcare practices, choices, and the context in which they lived. We organize our results by describing the characteristics of study participants followed by themes that emerged from the analysis: 1) perception of health and illness, 2) healthcare decision pathways, 3) negotiation between the environment and self in the emergence of self-reliance, and 4) efficiency and authenticity as a means to choose healthcare approach. The themes describe the interaction of various factors in the emergence of self-reliance.

### Characteristics of the study participants

Participants belonged to rural, tribal, and peri-urban contexts. The age range of participants was between 30 years to 80 years. Among the study participants, 19 (∼63%) were men and 11 (∼37%) were women. The majority of study participants, 20 (∼67%), had no schooling. All the study participants belonged to the Hindu religion. Within the Hindu religion, there were different caste categories (Table 1. Most participants, 20 (∼70%), were engaged in agriculture or daily wage work.

### Participants’ perception of health and illness

Health definitions of study participants ranged from being in a state of absence of disease to being happy and satisfied. The study participants gave importance to personal hygiene and food habits for maintaining their health. They considered health as a part of life’s needs. Their explanation of health was related to environmental factors, food habits, and lifestyles. Such an understanding of health was similar across rural, tribal, and peri-urban populations.

Terms used for defining health by the participants were *absence from the disease, being happy, being satisfied*, and *being pure*. We could interpret that the definition of health was based on an individual’s needs and where an individual lived. For instance, a participant from a periurban area defined health as the absence of a disease and being satisfied. When probed further, this was explained as an everyday readjustment to the demands of life. This struggle focused on their lived body and their lifestyle changes to sustain their health. The following statements by participants illustrate the same:

“*Health means to be pure. This is the village environment, it is pure. We don’t go to the city a lot. We work a lot in the field. Both men and women work hard. For us, diseases come rarely because we eat healthy food and breathe fresh air*.” (X6, Male, 51 years, farmer, rural area)

“*Health means being free from disease. We are in the city, the food we take is mixed with chemicals, and we never know what enters our bodies. Health is a gift for us. Our lives were different in our days, and we adjusted to the new changes and led. There is more stress here, but if we are satisfied, we can be healthy*.” (X9, Male, 62 years, farmer, peri-urban area)

Defining health was also understood as an opportunity to evaluate their (own) lives. Participants reflected into their past and compared their current lifestyle, habits, food they were eating, and physical activity they were engaged in. This comparison sometimes involved feelings of disappointment at the transition and acceptance of a new lifestyle. The following statement by a participant illustrates this:

“*These days food is not prepared using the chulla (ancient cooking method), earlier we used to use river water. Now we drink tap water, and it is not good water. We drink water and face health problems. If you keep water in a vessel, you will come to know how dirty it is. Our activity was our work from morning to evening; now, everything is machine-based. We were healthier since we were working more; now we are adjusting to this new life*.” (X8, Male, 70 years, farmer, rural area)

During the comparison process, study participants were able to reflect on their lives and define health in relation to their life experiences. This self-reflection provided a new broader perspective of their life rather than health alone. They mentioned improved technology, increased health services, and other new developments as an opportunity to enhance their well-being. This newer perspective came without compromising their traditions and beliefs. A participant expressed this as follows:

“*See, earlier we were not watching TV. Now we get all the information from the TV itself, which is very nice. Schools have come, and giving our children a good education is good, but whatever we watch, we should follow our tradition. For example, we worship God, which should continue, and we can’t leave just because we are watching TV. We should not get influenced by it*.” (X15, Female, 65 years, daily wage worker, tribal)

Thus, the comparative reflection of their past and their present gave hope and a better feeling to some participants. However, this always happened with the existential struggle between how they ought to live and how they actually live. Health for many of them was connected with their life (and way of living). Wherever they were able to do so, participants expressed enjoyment over their adaptability to the situation to reduce uncertainty and vulnerability. This adaptability created a positive feeling among them and brought a new perspective on their life. Such adaptability revealed the importance of the ability to make informed decisions in regard to their healthcare approach and the origin of such adaptability within how one perceives health and illness.

### Pathways of healthcare decisions

**Figure 2:**
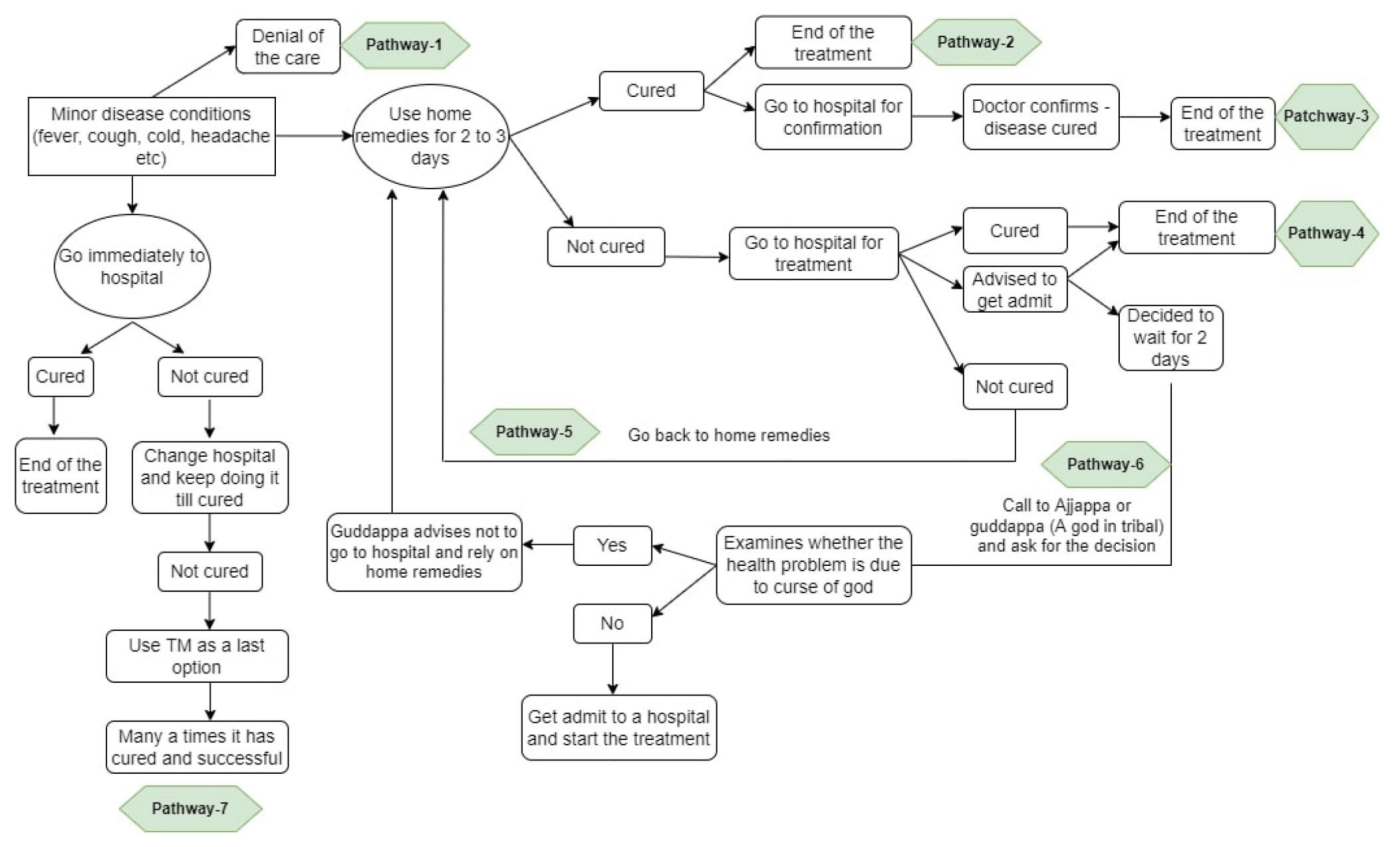
Healthcare approaches adopted by study participants.

The choice of a particular healthcare pathway was influenced by how participants perceived their health and illness. We identified seven patterns of pathways for healthcare decisions followed by study participants (Figure 2). For minor ailments, individuals commonly begin with home remedies for two to three days. If symptoms persist, they sought hospital treatment, consult traditional healers, or rely on spiritual mediums such as *Ajjappa* or *Guddappa* for guidance. Some participants move between hospitals, traditional medicine, and home-based care depending on the outcome, while others use traditional medicine as a last resort. These pathways reflect a dynamic negotiation between self-care, institutional healthcare, and spiritual practices, highlighting the interplay of tradition and modernity in health-seeking behavior. Healthcare pathways (Figure 2) adopted by the participants were most often trial and error. On a few occasions, participants were also able to make informed decisions (decisions emerging out of health information). For instance, presence of a family member knowing about or with experience of use of medicines (modern or traditional), elders known for their TM practice, a healer in the community, the presence of a local organization working on health, or a self-help group in the community were more likely to facilitate people in their health decisions.

While there were different sources for accessing information and health services, the healthcare pathways of participants were also strongly influenced by their customs, traditions, culture, and beliefs. Pathway-6 in Figure 2 illustrates how culture and beliefs influence healthcare decisions. Study participant (X19, Female, 40 year, Anganwadi helper, tribal area) expressed this as:

“*There is a person, sir. He is the head of our community. When we want to talk to God, we ask them to come, and he comes by wearing our dress and calls God in his body. They see the person and see if the disease is of medicine or God. If they say they should not go to the hospital, then we don’t go. Once God tells, it will cure. We have a belief that it will cure itself without the hospital. It has never failed, sir*.”

The complexity of healthcare decisions was evident in the community. This complexity further increased when healthcare decisions were made of panic, anxiety, or due to firmly rooted cultural and spiritual beliefs.

### Negotiation between the environment and the self

**Figure 3:**
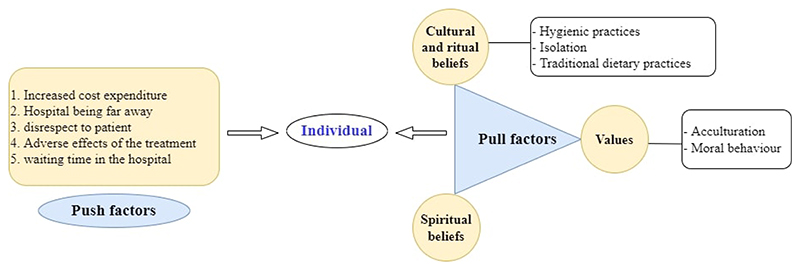
Push and pull factors of healthcare approaches.

In this study, the environment refers to the setting where an individual lives, the availability of health services, and the social context. The component of the self here denotes an individual’s beliefs, cultural and religious practices, and their beliefs around healthcare. Healthcare practices including TM practice were the result of push and pull factors (Figure 3). Push factors typically were challenges in relation to the health services and systems (inaccessibility, increased healthcare cost, and non-availability) and the pull factors were typically at the level of cultural values and beliefs relevant to healthcare practice. Participants expressed helplessness if their healthcare decision was due to push factors. However, positive feeling and confidence were witnessed if the healthcare decision was in alignment with the pull factors (culture and religious practices, spiritual practices, and belief system). This confidence and positive feeling impacted on their overall well-being. The tensions between push and pull factors were resolved through the emergence of a negotiation ability for an individual to redefine their health and readjust to the new transition. Participants expressed their transition with life rather than health alone. Their explanation of transition while negotiating with the environment came with many compromises which were evident in their expression of helplessness as illustrated in this quote:

“*What else we can do?? This is our life; if the hospital is nearby, it helps us. Now it is not there; we can’t help. We have to adjust and use what is available to treat our health problem. After coming here, at least our kids are going to school, which is happiness. School is far, but the kids go by walk. Even if we tell anyone, nobody listens, sir, it is our life, and we struggle and continue to live the same way. This has been our*.” (X15, Female, 65 years, daily wage worker, tribal)

Specific to TM use, apart from their easy availability and accessibility, the synchronization of one’s values and beliefs with TM treatment was the primary reason for adopting TM practices. As shown in Figure 3, themes of acculturation and moral behavior emerged, indicates practices of self-care were framed within broader concepts of *dharma* (religious duty), *nyaya* (justice), and *neeti* (moral conduct) as TM practitioners highlight these as part of self-care. The use of the term *hasiru aushadi* (green medicine in Kannada) for the TM denoted a strong connection between their environment, TM, and beliefs. Study participant (X8, Male, 70 years, farmer, rural area) expressed this as follows:

“*If you take our hasiru aushadi (green medicine), you will never get the disease back. It is more powerful and easily available here only. English medicine cures the problem, but there is a reoccurrence. Now it has become problematic, but we are left with no option. For everything, we are dependent on English medicine*”

The usage of our *hasiru aushadi* (our green medicine) indicates a close connection with their immediate surroundings and a sort of regret towards the interference of newly introduced treatment (modern medicine) in their existing lifestyle. The new change towards modern healthcare was adopted as a conditional situation rather than a choice by the participants as seen in their expression.

Thus, an individual does not exhibit self-reliance in isolation; instead, it manifests in relation to their social environment. This negotiation with the environment provided new directions, hope, and necessitated adaptations to the healthcare decision process.

### Efficiency a nd a uthenticity a s a m eans t o c hoose a healthcare approach

One of the critical factors that influenced the health decision was the speed of recovery. Whether one took a tablet, TM, or any other mode of treatment, the preference was given to that modality that cured the health problem quickly. For participants, it was efficiency (the ability to achieve desired health outcomes i.e., getting cured from symptoms of a particular illness; illness here refers to the subjective experience of symptoms, suffering, and the meaning individuals attach to being unwell, shaped by cultural and social contexts). The preferred outcome could be pain relief, stoppage of symptoms, or complete cure of a disease that bothered them the most rather than authenticity (proof or evidence backed by science). The healthcare practice ranged from using a medicinal leaf to treat the wound to reaching a doctor for the treatment. Study participant (X3, Male, 50-year, farmer, rural area) expressed it as:

“*See, we don’t know when health problem comes, whenever it comes, we use it. That’s all. For example, while working in the field, if we have any wound, we just take tumbe soppu (a local medicinal plant), crush it, and apply it to the wound to prevent bleeding. If you continue using it for three days, the entire wound will disappear. While we know these practices as part of our lifestyle, we also believe in rural areas that if we consult a hospital at the right time, it will save a life. That’s how it is believed here. Whatever we follow, we want quick treatment and go back to work in quick time*.”

For participants, efficiency mattered the most. Trust toward a particular treatment developed based on earlier outcome. Experiential knowledge was considered an authentic form of knowledge. Verification of the authenticity was vague and complex mired in one’s beliefs. For instance, a treatment taken using TM was cross-verified by meeting a doctor; a spiritual belief that endorsed a denial of treatment was considered authentic. This variation was quite evident among the study participants. However, the efficiency of the treatment was valued over the authenticity of the treatment.

## Discussion

### Healthcare choice and self-reliance

The manifestation of self-reliance is complex, and it is socially constructed because of the influence of various factors from the macro-level (environment) to the micro (individual) level [3]. As evident in this study, the healthcare approaches (Figure 2) adopted by study participants resulted from push and pull factors (Figure 3). Therefore, accessibility and availability of resources while necessary, is not sufficient for the emergence of self-reliance; instead, there is a need to consider culture, values, traditions, and beliefs [59,60]. Such knowledge combined with cultural wisdom, moral behavior, and practical realities will need to be integrated into efforts for developing policies and programs that are seen as empowering and promoting self-reliance [28,35,59]. Participants’ expression of a health problem as an illness rather than a disease reflects the need to consider their broader perspective instead of biomedical understanding of a health problem alone. To develop autonomy and competency and to bring responsible behavior to an individual, it is essential to deeply understand their healthcare experience and journeys through being in their shoes and discuss solutions from their perspective by allowing participants to reflect on their health practices, choices and journeys [45,61,62]. Self-reliance could be enabled when we protect individuals’ autonomy by considering them as a part of the system and not merely as passive consumers of a given scientific or modern healthcare service that is being provided, how much so ever efficacious it may demonstrably be. Hence, the health care decision which contributes to the emergence of self-reliance should be shaped by taking different factors into consideration. The emergence of quotes such as *‘village environment, it is pure’, ‘in the city, the food we take is mixed with chemicals’, ‘everything is machine-based. We were healthier since we were working more, earlier we were not watching TV. …whatever we watch, we should follow our tradition…we can’t leave just because we are watching TV’* from participants reflects the ongoing tension between tradition and modernity in healthcare practices in the context of self-reliance in primary health care. While traditional systems remain deeply embedded in community life due to their accessibility, affordability, and cultural resonance [63], they face challenges from the dominance of biomedical approaches within a neoliberal market-driven health system [64]. In this study, self-reliance entails the capacity of individuals to make informed decisions, balancing the use of home remedies and traditional practices, their apparent safety and efficacy along with timely engagement with modern institutional healthcare. Empowerment lies not in unchecked self-medication, and self-care but in awareness of the strengths and limitations of different approaches, supported by structures such as community health workers [65], regulation of TM, and referral linkages. Thus, healthcare choice in the context of self-reliance is not about letting individuals exercise their agency, rather it should be enabled in the larger framework of health and wellbeing by allowing the informed practice of pluralistic healthcare approaches that strengthens both individual agency and systems responsibility, ensuring safety, equity, and cultural legitimacy [66].

### Health literacy and self-reliance go hand in hand

Health literacy continues to remain an essential component of individual and community empowerment. It is an essential element to enhance informed healthcare decisions to enable self-reliance of individuals and communities. The critical part of health education is to provide a credible and consistent source of information [67]. In this study, the diverse health knowledge acquired by study participants reflected the reasons for practicing different healthcare approaches. The search for efficiency over authenticity indicated an urgency of recovery without adequate information or knowledge about a particular healthcare approach. This decision could lead to immediate gratification without any long-term health benefits. For instance, an individual experiencing severe gastric pain may use easily accessible home remedies or over-the-counter analgesics, which provide temporary comfort but do not treat an underlying condition such as peptic ulcer disease. Treating a health problem with inadequate information might result in detrimental effects or delay in seeking proper healthcare [68,69]. Such self-care actions do not necessarily develop competencies among the people but could create a false feeling of empowerment.

Health literacy is even more critical when dealing with TM practices due to its issues concerning evidence-based practice. The source of information concerning TM exists in the form of oral knowledge transmission, a priest in the community, a healer, or elders in the community, as observed in this study. Therefore, the range of TM practices involved using simple home remedies, cultural traditions, spiritual beliefs, and performing rituals [68,70]. As evident in the results, pathway 6 (Figure 2) is a delicate and complex issue since it involves belief systems and cultural practices. Spiritual practices and rituals in TM can offer psychological relief, social reassurance, and culturally meaningful ways of addressing illness, and should not be dismissed outright [71,72]. At the same time, it is important to strengthen health literacy among practitioners and communities while advancing rigorous research on herbal and other TM systems to assess efficacy and safety [13,73,74]. Such a balanced approach helps preserve supportive practices while mitigating risks linked to misconceptions. Therefore, the goal of health literacy when dealing with TM should also be to address misconceptions and harmful beliefs [67,68,70].

The inconsistent information is also because of inadequate knowledge among healthcare providers. In the long run, this leads to a lack of trust between the healthcare provider and the patient. Globally and in India, public health programs are policies are being rolled out to empower communities and individuals by through health education delivered by community health workers to communities. However, monitoring health worker competencies, examining their own views and perspectives on healthcare decision-making, updating their curriculum based on the current needs, and capacity-building sessions for community health workers are often overlooked. Hence, this has resulted in a lack of credible information and uneven advancement. Therefore, the trial-and-error approach to treating an illness continues to exist among people and healthcare providers. Thus, health education is an essential element of self-reliance and continues to be an important area to tap into with innovative approaches to enhance the capacities and competencies of individuals, communities and of health workers.

### Conceptualization of self-reliance

**Figure 4:**
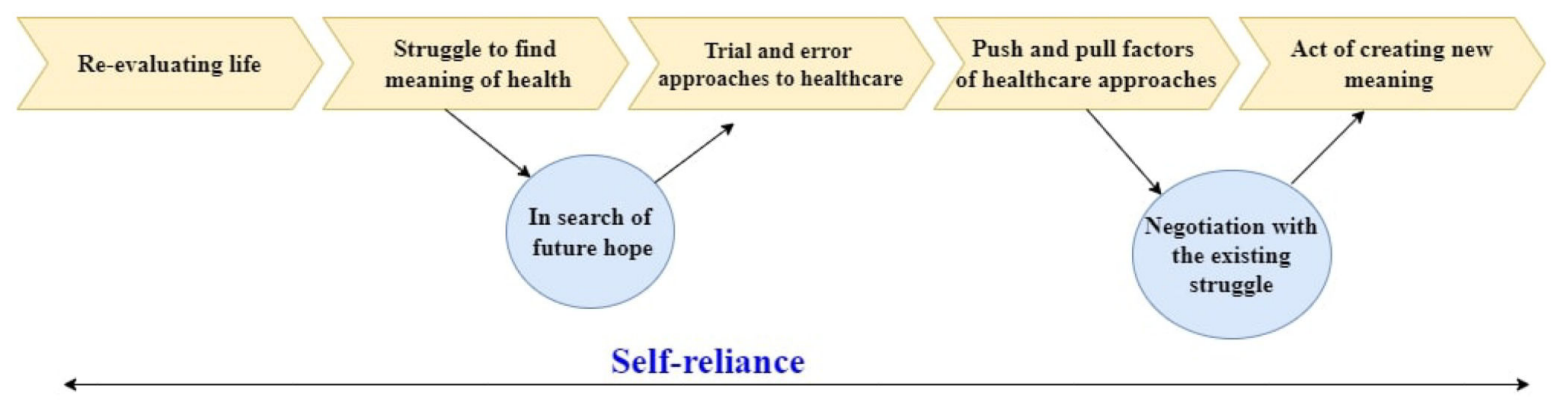
The process of meaning creation of self-reliance.

The underlying principle in participants’ understanding of health seems to be a negotiation between their bodily requirements and the environmental factors that influence them. Thus, this negotiation allowed participants to bring a new perspective to their health. These new perspectives sometimes were experienced as boring and uninspiring by the participants. However, this also provided a better meaning to their understanding of health (Figure 4). The awareness of various factors that influence their health perspective is a continuous process. Participants struggled to define their health with every new change in their context, or sometimes it was just moving with the flow of life. As expressed by study participants, the trial-and-error approach necessitated by the urgency to return to work or sometimes due to anxiety and panic (of the illness), and choice of healthcare was sometimes due to their perception of efficiency and authenticity of TM practices. In addition to this decision struggle, an environment with pluralistic healthcare choices added further confusion because of various push and pull factors. Healthcare decisions took place through a negotiation process within an individual between one’s beliefs, values, practices, and the presence of resources. Hence, this study does not claim a clear pathway for the manifestation of self-reliance but instead captures it as a process, dynamic, state of mind, and context-dependent. A state of self-reliance is determined by an individual’s felt autonomy, relatedness, confidence, and competency to make health decisions [61]. This state of mind is sustained as long as an individual’s autonomy and competency are protected. Our findings align with the studies that captured self-care as an existential struggle between the subjective understanding of the body and an external environment [46,75]. Thus, the meaning “creation act” comes from self-reflection of one’s thoughts and actions concerning the environment an individual is living in. The new meaning was enjoyed if that provided new hope and positive feelings; otherwise, the negotiation process was reconsidered, and the act of creating meaning continued. This self-reflection is vital for an individual to develop a new way of managing his health by developing a new competency. For individuals to become managers of their health, health systems could stimulate self-reflection. This approach, in turn, contributes to the decision-making ability of an individual about healthcare choice which is a key component of self-reliance. Based on the understanding of various factors from the synthesis of our study findings that influenced the emergence of self-reliance, we define self-reliance as ‘the ability of an individual to make informed health decisions to use local resources (formal and informal health services, and medicinal plants) considering social and cultural beliefs to achieve sustainable health behavior through which an individual achieves a state of autonomy, competent, and responsible towards his/her health that gives a sense of empowerment to an individual. Such a state of health behavior that is flexible and adaptable to a particular context is termed as self-reliance.’

### Policy implications of the study

*Ayushman Bharat* health and wellness program in India emphasizes self-care, informed choice, respectful care, people-centred care, and confidentiality in its policy guidelines [38]. This study re-emphasizes those principles by emphasising self-reliance of individuals and communities. AYUSH health and wellness centres emphasize using medicinal plants for primary healthcare in addition to self-care, informed choice, and people-centred care. However, the approach of AYUSH health and wellness again centred around an institutional-based approach. Although with well-designed strategies and objectives, the *Ayushman Bharat* program continues to face practical challenges of monitoring and conducting community-level activities due to manpower shortage [39]. This necessitates building capacities of the people to address the crisis of manpower for the utilization of services of the program. Keeping the principles of self-reliance, now is the time to reflect on how people need to get involved to make the Ayushman Bharat program successful in achieving its objectives of universal health coverage and empowering people to make informed choices. The AYUSH health and wellness program also emphasizes moral behaviour (*sadvrita*) for the well-being of people which aligns with the findings of this study where *dharma* (religion), *nyaya (justice), and* neeti* (moral conduct) are part of peoples’ self-care.

The definition of self-care by WHO does not mention the influence of contextual factors while acquiring abilities [76]. Also, the definition is more affirmative in its position that omits the flexible nature of an individual’s abilities in managing health consequences. In contrast, this study provided an understanding of self-reliance as flexible, dynamic, and context-specific. Self-reliance understanding helps in designing policy and practice guidelines that are comprehensive and applicable based on the contextual understanding of a health intervention while working with the people and communities.

Our findings resonate with the “Fourth-Tier” concept of the Indian health system, which recognizes individual and community self-reliance as a critical complement to primary, secondary, and tertiary care [77]. By highlighting people’s perspectives and local practices, this study conceptualized how self-reliance operates as an integral part of the health system and potentially be considered in health policy and program design.

### Strengths and limitations of the study

This study provided a detailed and in-depth description of the self-reliance phenomenon through the case study method focusing on a forested district in the Western Ghats biodiversity hotspot of India where TM practices are widespread. We also provided a contextual perspective of self-reliance by going in-depth and allowing people to self-reflect on their practices, beliefs, and healthcare choices. Understanding ‘the self’ in relation to the environment is essential knowledge to contribute to a people-centered health system, and our study contributes to the same.

Several limitations should however be considered. This study focused on a specific project implemented by TDU which limits the coverage of other contexts which could have provided alternate insights into self-reliance. Our study has heavily focused on the self-component and least on the environment component while conceptualizing self-reliance. A rival case (household not practicing TM) explanation was not included in the results, which we feel is a limitation. Although we covered rural, peri-urban, and tribal contexts to conceptualize self-reliance, the influence of gender, caste, and religion was not explored. This study did not examine either the socioeconomic determinants of health service access, including facility distribution, user fees, and affordability, which may have influenced care-seeking behaviors in the study setting. The majority of our study participants were more than 50-years-old although this selection was not intentional. When younger participants were asked to participate in the study, they mentioned that TM practices could be better captured by interviewing their parents and hence denied participating in the study. Neoliberal policies also emphasize autonomy and reducing the interference of state agencies. This study did not discuss those deliberations while explaining self-reliance. Future studies should investigate how these policies shape the framing and practice of self-reliance in healthcare systems. Finally, as the data collection period overlapped with COVID-19 pandemic, it might have influenced our study findings. Future studies need to take into consideration these limitations while conceptualizing self-reliance since contextual factors determine healthcare choice, and practices of people.

### Conclusion and recommendations

The present study provided an understanding of self-reliance within the health system from people’s perspectives and embodied experiences of integrative healthcare practices. Providing an opportunity and autonomy for individuals to self-reflect on their thoughts and actions is essential to navigating a solution to the existing struggle to choose a healthcare approach or even to understand one’s health and well-being. This autonomy helps to develop one’s competencies to enable self-reliance. While many self-treatment practices exist in the community including TM, the current health education strategy is limited to the prevention and promotion aspects of primary health care. Identifying best practices based on the evidence is a way to promote such practices to address minor health conditions and reduce the burden on the health system. Providing adequate and consistent information to people is crucial to enhancing the health literacy of people and communities. This literacy further builds peoples’ competency and confidence in their practice. Protecting autonomy while empowering individuals and communities comes from understanding their cultural traditions and beliefs. This understanding helps to develop strategies that are context-specific and people-centric. Thus, self-reliance can’t be understood as self alone; one must meaningfully understand it in relation to the environment and social context. Capturing local views and peoples’ perspectives help policymakers to understand the gap in relation to scientific perspective and local knowledge and thereby brings solutions that connect both local views and science by carefully locating them in the context of the health system.

With the increasing burden of non-communicable diseases, the need for empowering people to be responsible for their health is more crucial than ever before. In this direction, creating self-reliant individuals and communities is essential for achieving a people-centred health system. People-centred health systems target to achieve patient satisfaction and increased responsiveness to care by meeting non-medical expectations (respect for people by protecting their dignity, confidentiality, and autonomy). The integration of education, family involvement, self-management, and counselling into healthcare enhances the competencies and skills of people that in turn enhances self-reliance in primary healthcare among families and communities.

Future studies need to position an explanation of self-reliance considering neoliberal policies so that clarity of understanding self-reliance is provided in the context of such policies. Although the study provided an understanding of the interaction of various factors in the emergence of self-reliance, the study recommends developing a self-reliance framework that provides a systemic understanding of factors at different levels that could guide in designing health interventions to enable self-reliance in primary healthcare. A follow-up study capturing the changes in perspectives of younger generations about TM healthcare practices, and the loss of this knowledge over time, would be useful to further enhance our understanding of changes in self-reliance in relation to TM.

## Figures and Tables

**Table 1:** Characteristics of study participants.

Name	Village	Age (yrs.)	Sex	Education	Religion	Caste	Occupation	Context
X1	A1	52	F	No schooling	Hindu	Kuruba Gowda	Daily wage worker	Rural
X2	A1	40	M	9th std	Hindu	Lingayat	Agriculture	Rural
X3	A2	50	M	10th Std	Hindu	Okkaliga Gowda	Agriculture/Business	Rural
X4	A2	72	F	No schooling	Hindu	SC	Daily wage worker	Rural
X5	A2	50	M	No schooling	Hindu	Okkaliga Gowda	Daily wage worker	Rural
X6	A3	51	M	3rd std	Hindu	Okkaliga Gowda	Agriculture	Rural
X7	A4	40	F	7th std	Hindu	Lingayat	Homemaker	Rural
X8	A5	70	M	No schooling	Hindu	Okkaliga Gowda	Agriculture	Rural
X9	A6	62	M	7th std	Hindu	SC	Agriculture	Peri-urban
X10	A7	60	M	No schooling	Hindu	Jenu Kuruba	Agriculture	Tribal
X11	A8	51	M	No schooling	Hindu	Jenu Kuruba	Agriculture	Tribal
X12	A8	68	F	No schooling	Hindu	Jenu Kuruba	Daily wage worker	Tribal
X13	A9	50	F	No schooling	Hindu	Erava	Homemaker	Tribal
X14	A9	38	F	No schooling	Hindu	Erava	Homemaker	Tribal
X15	A10	65	F	No schooling	Hindu	Betta Kuruba	Daily wage worker	Tribal
X16	A6	36	M	10th Std	Hindu	Lingayat	Business	Peri-urban
X17	A11	52	M	10th Std	Hindu	Lingayat	Agriculture	Rural
X18	A12	65	M	No schooling	Hindu	Lingayat	Agriculture	Rural
X19	A13	40	F	No schooling	Hindu	Betta Kuruba	Anganwadi Helper	Tribal
X20	A14	78	M	No schooling	Hindu	Jenu Kuruba	Daily wage worker	Tribal
X21	A15	70	M	1st std	Hindu	Lingayat	Agriculture	Rural
X22	A16	57	M	No schooling	Hindu	Shettaru (Kumbar)	Agriculture	Rural
X23	A17	60	M	No schooling	Hindu	Jenu Kuruba	Daily wage worker	Tribal
X24	A18	72	M	10th Std	Hindu	Okkaliga Gowda	Agriculture	Rural
X25	A18	42	M	No schooling	Hindu	Soliga	Daily wage worker	Tribal
X26	A18	32	F	No schooling	Hindu	Okkaliga Gowda	Agriculture	Rural
X27	A19	36	M	No schooling	Hindu	Jenu Kuruba	Daily wage worker	Tribal
X28	A20	77	M	No schooling	Hindu	Okkaliga Gowda	Agriculture	Rural
X29	A21	32	M	10th Std	Hindu	Kadu Kuruba	Medicine preparation at the Ayurveda centre	Tribal
X30	A22	76	F	No schooling	Hindu	SC	Daily wage worker	Rural

## Data Availability

The datasets generated for this study can be found in the figshare database. https://figshare.com/articles/dataset/Self-reliance_study_HD_Kote_Shivabs_rar/ 20525694.
